# Mitochondrial form and function in hair cells

**DOI:** 10.1016/j.heares.2022.108660

**Published:** 2023-02

**Authors:** James D.B. O'Sullivan, Anwen Bullen, Zoë F. Mann

**Affiliations:** a Centre for Craniofacial and Regenerative Biology, Faculty of Dentistry, Oral, Craniofacial Sciences, King's College London, London SE1 9RT, U.K; b UCL Ear Institute, University College London, London WC1×8EE, U.K

**Keywords:** Mitochondria, Metabolism, Mitochondrial cristae, Ca2+buffering, Synaptic transmission oxidative stress, Hearing loss, Development

## Abstract

Hair cells (HCs) are specialised sensory receptors residing in the neurosensory epithelia of inner ear sense organs. The precise morphological and physiological properties of HCs allow us to perceive sound and interact with the world around us. Mitochondria play a significant role in normal HC function and are also intricately involved in HC death. They generate ATP essential for sustaining the activity of ion pumps, Ca^2+^ transporters and the integrity of the stereociliary bundle during transduction as well as regulating cytosolic calcium homoeostasis during synaptic transmission. Advances in imaging techniques have allowed us to study mitochondrial populations throughout the HC, and how they interact with other organelles. These analyses have identified distinct mitochondrial populations between the apical and basolateral portions of the HC, in which mitochondrial morphology appears determined by the physiological processes in the different cellular compartments. Studies in HCs across species show that ototoxic agents, ageing and noise damage directly impact mitochondrial structure and function resulting in HC death. Deciphering the molecular mechanisms underlying this mitochondrial sensitivity, and how their morphology relates to their function during HC death, requires that we first understand this relationship in the context of normal HC function.

## Highlights

• Mitochondria play a significant role in function and death of auditory hair cells.
• Advances in imaging have revealed new aspects of mitochondrial biology.
• Spatially and functionally discrete mitochondrial populations support sensory function.
• The relationship between mitochondrial structure and function is poorly understood.
• Future work should focus on the role of mitochondria-organelle interactions.

## 1 Introduction

Hearing loss is a growing and unmet medical need in the general population (Agrawal et al., 2009; Haile et al., 2021), which when permanent is accompanied by the loss of sensory hair cells (HCs) (Nadol et al., 2015; Nordmann et al., 2000; Suga and Lindsay, 1976; Wu et al., 2020). These cells are highly specialised mechanoreceptors residing in the inner ear sensory epithelia. In mammals, HCs of both auditory and vestibular epithelia cannot regenerate after damage and, as there are no known therapies to replace them once lost, the resulting hearing loss or balance dysfunction is permanent. By recapitulating aspects of early inner ear development, it is now possible to generate HCs in organoid-type models using both human and mouse stem cells. Although organoid-derived HCs display many features of those in a normal ear, they are not functionally viable long-term and arrest developmentally at late embryonic stages (Koehler et al., 2017, 2013; van der Valk et al., 2021)***.*** In attempts to address this problem, significant effort has been directed to understanding the molecular mechanisms and cell biology underlying HC formation, physiological function, and degeneration.

The processes associated with detection and analysis of sound by HCs (mechanotransduction) are metabolically demanding (Lesus et al., 2019). A plentiful supply of energy in the form of cellular ATP is therefore required for optimal HC function. Mitochondria have come under scrutiny both as key regulators of HC physiology, and as targets vulnerable to the ototoxic agents and damaging noise stimuli that induce HC death (Lesus et al., 2019). In all tissues the specific morphology and cellular distribution of mitochondria are highly correlated with the specific functions that particular cell types perform (Glancy et al., 2020)***.*** The formation and remodelling of organised mitochondrial network architecture through fusion and fission also plays a key role in shaping the cell fate decisions during differentiation in multiple cell types (Bahat and Gross, 2019; Iwata et al., 2020). The advent of high-resolution volume electron microscopy imaging techniques such as Serial Blockface Scanning Electron Microscopy (SBEM) and Electron Tomography (ET) have made it possible to study mitochondrial distribution and ultrastructure in detail across a variety of cell types including HCs (Peddie and Collinson, 2014). These analyses have provided insight into their regulatory role in normal HC function (Bullen et al., 2015; Lesus et al., 2017). Furthermore, work in transgenic mouse and zebrafish lines has shed light on the role mitochondria play in mechanotransduction and synaptic transmission at the HC ribbon synapse, and how their activity is adversely affected under pathophysiological conditions (Holmgren and Sheets, 2021; Manikandan et al., 2021; Pickett et al., 2018; Wong et al., 2019).

In this review we compile recent evidence highlighting the central role that mitochondria play in regulating aspects of HC function in inner ear sensory epithelia and discuss what is known about mitochondrial function and cellular metabolism in cells of the inner ear across different species. We compare differences in mitochondrial localisation between HC subtypes and examine the functional consequences of these distribution patterns. We finally summarise studies highlighting their role in mechanosensory transduction, synaptic transmission as well as in drug- or chemical- induced hair cell death (ototoxicity) and noise damage.

## 2 Models to study mitochondrial and metabolic function in cells of the auditory system

In all the sensory epithelia of the inner ear, HCs exist within a cellular mosaic, alongside their auxiliary glial-like supporting cells (SCs). The sense organs of the vestibular system are the maculae of the utricle and saccule and the cristae of the semi-circular canals, not to be confused with the substructural cristae of a mitochondrion. The auditory epithelia are the organ of Corti in mammals, and the basilar papilla (BP) in birds, lizards and reptiles (Hirokawa, 1978; Manley and Clack, 2004; Pfaff et al., 2019)***.*** Differences in the structure and organisation of auditory epithelia between different orders of vertebrates lead to distinct mechanisms of auditory processing. The numerous models of auditory epithelia provide a variety of contexts in which to study the cell biology of the resident cell types.

### 2.1 Mammals

**Fig. 1:**
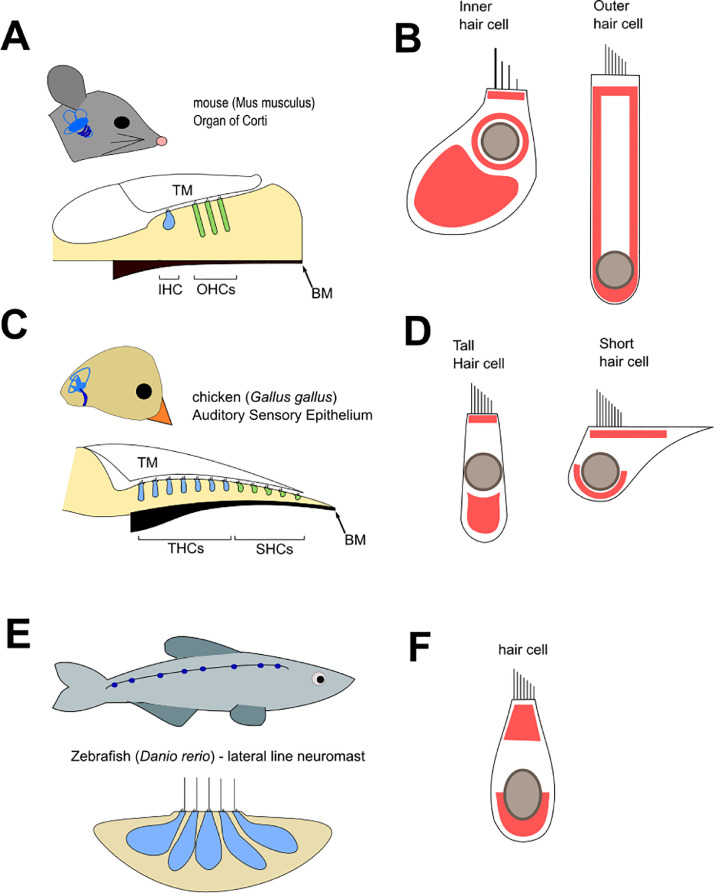
**The arrangement of sensory epithelia and hair cell mitochondria in the mouse, chicken and zebrafish:** A) The mouse cochlea is divided into inner hair cells (IHC; blue) and outer hair cells (OHCs; green). The sensory epithelium is covered by the tectorial membrane (TM), which contacts the stereocilia of the HCs. The basilar membrane (BM) lies underneath the epithelium. B) Representation of mammalian hair cells. The IHCs have an apical and basal population of mitochondria (indicated by red areas), in addition to a population of mitochondria surrounding the nucleus (Bullen et al., 2015). OHCs have apical and basal mitochondrial populations, in addition to mitochondria in close proximity to the lateral cell membranes (Weaver and Schweitzer, 1994). C) The chicken cochlea is divided into tall hair cells (THCs; blue), and short hair cells (SHCs; green). D) Representation of avian hair cells. Electron microscopy (Hirokawa, 1978) shows populations of mitochondria (blue) both above and below the nucleus. E) The lateral line of zebrafish is punctuated by the sensory neuromasts. Each neuromast contains a circular cluster of HCs. F) Representation of a HC from the neuromast. Mitochondria are visible above and beneath the cell nucleus (Laurà et al., 2018).

The organ of Corti comprises a single row of inner hair cells (IHCs) and three rows of outer hair cells (OHCs) (Fig. 1A). IHCs and OHCs are morphologically and functionally distinct, with the former being used primarily for frequency detection and the latter providing signal amplification and enhancing frequency selection (Ulfendahl and Flock, 1998; Ashmore 1987, Gale & Ashmore 1997). OHCs undergo rapid length changes in response to transduction currents, a process driven by the motor protein prestin (Ashmore, 1987; Dallos, 2008; Dallos et al., 1991; Géléoc and Holt, 2003; Holley and Ashmore, 1988; Liberman et al., 2002). These changes in OHC length determine the frequency sensitivity and selectivity of the system. In the mammalian cochlea, the basilar membrane (BM) is the primary frequency analyser (Fig. 1A). Pure tone stimuli enter the ear and produce local vibrations of the basilar membrane at different positions along the basal-to-apical (tonotopic) axis. Mechanical tuning in the cochlea relies on graded differences in the passive properties of the acellular basement-membrane like basilar membrane (BM), underlying the cells of the organ of Corti, and of the acellular tectorial membrane (TM), which lies across the apical surface of the organ of Corti. The composition and mechanical properties of both these acellular structures show tonotopic differences (Mao et al., 2020; Richter et al., 2007; Tani et al., 2021; Teudt and Richter, 2014). Differences in the passive BM properties between the cochlear base and apex allow specific frequencies to elicit maximal responses in HCs located at corresponding positions along the cochlea (tonotopy) (Johnstone et al., 1970; Robertson and Manley, 1974; Russell and Sellick, 1977). The motion produced from sound vibrations is detected by the sensory HCs and transmitted as precisely timed and synchronized firing via single auditory nerve fibres to the cortex (Fettiplace, 2017).

### 2.2 Birds and lower vertebrates

In the BP, frequency tuning is achieved mainly through intrinsic filtering of sound stimuli within the HCs themselves (Crawford and Fettiplace, 1981; Fettiplace and Fuchs, 1999; Mann and Kelley, 2011; Mao et al., 2020)***.*** Graded differences in the expression and gating kinetics of ion channels along the tonotopic axis (Fettiplace and Fuchs, 1999; Frucht et al., 2011; Fuchs et al., 1990; Fuchs and Evans, 1990; Miranda-Rottmann et al., 2010) allow HCs to resonate electrically in response to specific frequencies (Art and Fettiplace, 1987; Crawford and Fettiplace, 1981; Fettiplace and Fuchs, 1999; Fuchs et al., 1988)***.*** In the BP HCs with varying morphologies are arrayed along the long proximal-to-distal and across the abneural-neural axes (Fig. 1C) (Cotanche and Kaiser, 2010; Tilney et al., 1992, 1986; Tilney and Saunders, 1983). Tall hair cells (THCs) occupy the neural side, and short hair cells (SHCs) the abneural side (Hirokawa, 1978). These two populations of cells are named based on their tall and squat morphology, respectively (Fig. 1). Based mainly on similarities in patterns of innervation (Fischer, 1992; Tan et al., 2013), it is thought that THCs are analogous to mammalian IHCs, whilst SHCs are analogous to OHCs. This neural organisation is conserved across multiple avian species (Takasaka and Smith, 1971; Umemoto et al., 1993).

### 2.3 Zebrafish

The zebrafish has also proved an important model for investigating HC development, physiology, and death. Like other species of fish (Montgomery et al., 2000) and some cephalopods (Budelmann and Bleckmann, 1988), the zebrafish possesses a series of superficial sensory neuromasts at intervals along its lateral side (Fig. 1E). Each neuromast contains a roughly circular aggregate of HCs which detect local water currents. There are notable structural differences between the neuromast and amniote auditory sensory epithelia. For instance, neuromasts lack the tonotopy and HC subtype specialisation seen in mammals and lower vertebrates and there is no evidence of active HC motility despite presence of prestin orthologs (Tan et al., 2011). However, many functional features of HCs are conserved between zebrafish and amniotes. Given the positioning of the neuromast on the surface of the animal HCs can be readily exposed to pharmacological agents, and are easily accessible for in vivo imaging (Holmgren and Sheets, 2021). The zebrafish therefore provides an ideal system in which to study and analyse the dynamics, physiology and morphology of mitochondria throughout the HC.

## 3 Mitochondrial structure and its relationship to function

The structural study of mitochondria in HCs has undergone a recent and significant resurgence, following the development of new imaging techniques. 3D volume electron microscopy (Peddie and Collinson, 2014) and the ever more high-resolution light microscopy techniques, particularly in the fields of live cell imaging and cryo-fixation of tissues, permit large-scale three-dimensional analysis of complex tissues like the cochlea. The complex relationship between the structure and function of sub-cellular organelles is vital to the highly specific functional needs of HCs. The opportunities to examine this important aspect of HC biology in unprecedented detail have never been better and characterising these relationships and their regulation in HCs is vital in understanding their physiology and dysfunction. Over recent years it has become clear that mitochondrial ultrastructure and cellular distribution determine their activity and functional roles in the cell. The dynamic nature of mitochondrial morphology, and how their size, number and positioning within the cell is controlled has provided valuable insights into how mitochondria support and define cellular function.

### 3.1 Mitochondrial distribution

Mitochondria are more than simply static ‘energy factories,’ and the study of how mitochondrial populations are arranged in the cytosol, and how they move through the cell, is key for understanding how cells utilise their mitochondrial population. Mitochondria at different cellular sites have differing morphologies and biochemical properties in turn allowing them to regulate distinct aspects of the cellular function. Mitochondria in one area may be primarily concerned with ATP generation, while those in other areas regulate the spatiotemporal dynamics of cytosolic calcium signals or modulation of cellular functions through reactive oxygen species (ROS) generation (Glancy et al., 2020). There is also evidence of communication across inter-mitochondrial junctions, reminiscent of bacterial quorum sensing, a system of chemical signals produced by individual bacteria in a colony which coordinate colony wide functions (Picard et al., 2015; Vernay et al., 2017).

Mitochondria are motile, and their movement is predominantly regulated by microtubule-based kinesin/ dynein motors, although mitochondria have also been observed to travel along actin filaments by myosin motors in budding yeast (Frederick and Shaw, 2007). Mitochondrial movement has been shown to heavily rely on the GTPases Miro-1 and Miro-2 in mammalian cells, although some movement is preserved even when both proteins are absent (López-Doménech et al., 2018). In neuronal cells mitochondria move long distances travelling from the cell body to distantly located synaptic sites. This movement is thought to maintain energy homoeostasis and aid calcium signalling in long axons, and is defective in several neurological diseases; Alzheimer's, Huntingdon's, and Parkinson's are all associated with disrupted mitochondrial movement (López-Doménech et al., 2016; Lovas and Wang, 2013; Park et al., 2018; Flannery and Trushina, 2019). Recognition of the importance of mitochondrial distribution and ultrastructure has furthered understanding of how highly specialized auditory HCs perform their function.

### 3.2 Mitochondrial shape

**Fig. 2:**
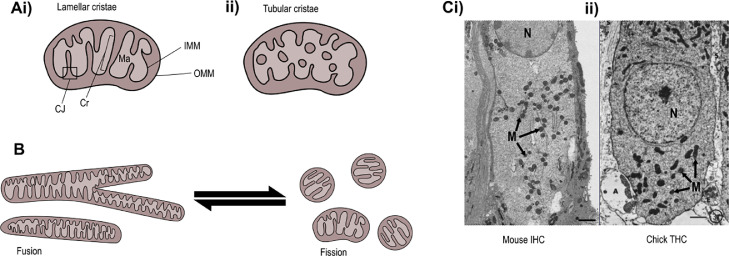
**Ultrastructure and function of mitochondria.** Ai) Mitochondria possess an inner mitochondrial membrane (IMM) and an outer mitochondrial membrane (OMM) which separate the organellar compartments required for ATP generation. The IMM houses the electron transport chain and is folded into cristae (Cr), which are lamellar structures in most cells, although they may have a more tubular structure in some cases (Aii). Each crista can act as an independent energy generating unit with its own membrane potential, and this compartmentalisation is maintained by the crista junctions (CJ) at the base of each crista (Wolf et al., 2019). The Kreb's cycle enzymes are housed in the mitochondrial matrix (Ma). B) All mitochondria exist in an equilibrium between elongated, interconnected networks and smaller, fragmented or even spherical individual unit. The balance between these two states is determined by the incidence of fusion and fission events. C) electron micrographs showing HC nuclei (N) and mitochondria (M). Mouse IHCs (i) and their analogues in chicken, the THCs (ii), exhibit different mitochondrial morphologies. Image of the THC was reproduced with permission from the publisher after Hirokawa et al. (1978), and the IHC image is from the author's (AB) original imaging.

The definition of mitochondrial shape has been attributed to two main factors, mitochondrial fission and fusion (Fig. 2B). Elongated tubular networks generated by fusion of several individual mitochondria allow for distribution of matrix components and the exchange of mitochondrial DNA (mtDNA). This is essential to maintain active oxidative phosphorylation (OXPHOS) in mitochondria (Otera and Mihara, 2011) and for the maintenance of mtDNA, with loss of fusion correlated with severe mtDNA depletion (Ramos et al., 2019). Fission promotes smaller, more discrete, spherical mitochondria. Although mitochondrial fission (or fragmentation) is often linked to cell dysfunction, cell stress and death, it is also required for mitochondrial mobility, quality control and replication during cell division (Zemirli et al., 2018). Mitochondrial shape changes can also occur through mechanisms that are independent of fission and fusion such as mitochondrial shape change (MiST), a process driven by cytosolic calcium sensing, but distinct from fission and mitochondrial swelling (Nemani et al., 2018). The morphology of mitochondria varies between tissues and across cell types, for example the numerous, large, elongated mitochondria found in the ion-pumping marginal cells of the stria vascularis, and the smaller more spherical mitochondria found at lower density in the intermediate cells of the same tissue (Hinojosa and Rodriguez-Echandia, 1966). Small spherical mitochondria are found in the liver, while in brain white matter mitochondria exist as elongated tubular structures, and in skeletal muscle, mitochondria reside as highly elongated and branched networks (reticulum). The morphology of mitochondria in these tissues also changes with age and in response to cellular stress (Glancy et al., 2020)**.**

3D electron microscopic examination has shown that adult mouse IHCs contain a majority of discrete, spheroid mitochondria (Bullen et al., 2015; Liu et al., 2021). However, little is known about mitochondrial shape in aged HCs, nor do we fully understand the mitochondrial dynamics associated with normal HC function, compared to what is known about mitochondrial structure and dynamics in other neuronal cell types. In addition, there appear to be taxon-specific differences in mitochondrial shape. Unlike their murine counterparts, chick THCs and SHCs appear to have elongated, non-spheroid mitochondria when examined using TEM (Hirokawa, 1978). In the zebrafish lateral line, the fusion/fission state is less clear in some published electron micrographs (Dow et al., 2018; Laurà et al., 2018), with otherwise highly informative live imaging experiments lacking sufficient spatial resolution to provide further clarity on this specific issue (Pickett et al., 2018). However, unpublished work specifically investigating mitochondrial morphology in the zebrafish lateral line HCs using SBF-SEM has revealed that apical mitochondria are smaller and more discrete, whilst those at the base of the cell form a larger reticular network (McQuate et al., 2022). Disruption of network architecture results in a reduced mitochondrial response to mechanical stimulation of the HC. In addition, mechanotransduction and synaptic activity appear to be important in establishing network architecture.

In other cell types, the mechanisms regulating mitochondrial fission and fusion events are often linked to other cellular events, including cytosolic calcium signalling (Ji et al., 2017), membrane depolarisation (Fung et al., 2019) and mechanosensation (Feng and Kornmann, 2018). In turn, changes in mitochondrial shape can have significant effects on cellular functions; particularly relevant to cells of the inner ear are the role of mitochondrial fission in controlling the spatial and temporal dynamics of mitochondrial calcium responses (and by extension the effects of cellular calcium signals) (Szabadkai et al., 2004), and the role of increased mitochondrial fission in increased ROS levels across several cell types which can be reversed by induction of fusion (Huang et al., 2016; Yu et al., 2019, 2006).

### 3.3 Mitochondrial distribution and function in auditory HCs

The specialised functions of HCs to sense mechanical vibration from sound or movement and to transduce these vibrations into electrochemical signals suggests that HCs will have both significant energy demands and require mitochondria in different parts of the cell to perform different functional roles to assure efficient transduction. Indeed, this is the case (Fig. 1B, D, F). The projecting bundles of stereocilia emerge from the apical surface of each cell and are anchored in place by the actin-rich cuticular plate (Engström and Engström, 1978), a structure located just beneath the HC apical membrane. In addition to the large number of ion channels, the basolateral membrane of the HC hosts the synapses for transmitting auditory signals or receiving efferent input. Mammalian IHCs possess synaptic ribbons close to their afferent connections which promote rapid and sustained neurotransmitter release (Nouvian et al., 2006; Safieddine et al., 2012). These structures are composed of the protein Ribeye, and their primary role is to regulate trafficking and fusion of vesicles in the presynaptic terminal, permitting high fidelity transmission of sound stimuli. This process is highly regulated by ATP and intracellular Ca^2+^ (Frank et al., 2009; Heidelberger et al., 2002; Snellman et al., 2011). The basal and apical portions of HCs are linked by a range of membranous networks (Bullen et al., 2015; Perkins et al., 2020). As shown in Fig. 1, the distribution of mitochondria varies between HC types and across species, however some commonalities can be observed; the presence of a mitochondrial population that directly underlies the stereocilia, and a second infranuclear population in the region of the synapses. This subcuticular and basolateral mitochondrial localisation is broadly shared across species, and has been documented in electron micrographs from mouse, human, and chick auditory HCs, as well as zebrafish HCs of the lateral line (Hirokawa, 1978; Bullen et al., 2015; Laurà et al., 2018; Liu et al., 2020). Both cochlear and vestibular HCs have specialised subsets of mitochondria in their apical regions, with large subcuticular mitochondria surrounding the Hensen bodies in OHCs (Lesus et al., 2019). The Hensen bodies are roughly spherical structures consisting of membranes either in lamellar or clustered vesicular formation (Spicer et al., 1998). They are derived from the smooth endoplasmic reticulum and are located basally in relation to the cuticular plate. Vestibular HCs contain the striated organelle just beneath the cuticular plate, involved in adaptation of the receptor potential, and surrounded by a population of large mitochondria. It has been suggested that these mitochondria may buffer Ca^2+^ and supply necessary ATP for the contractile apparatus of the cell (Vranceanu et al., 2012). The role of mitochondria in HC Ca^2+^ homoeostasis and regulation of plasma membrane Ca^2+^ ATPase (PMCA) activity will be discussed in Section 6.2.

**Fig. 3:**
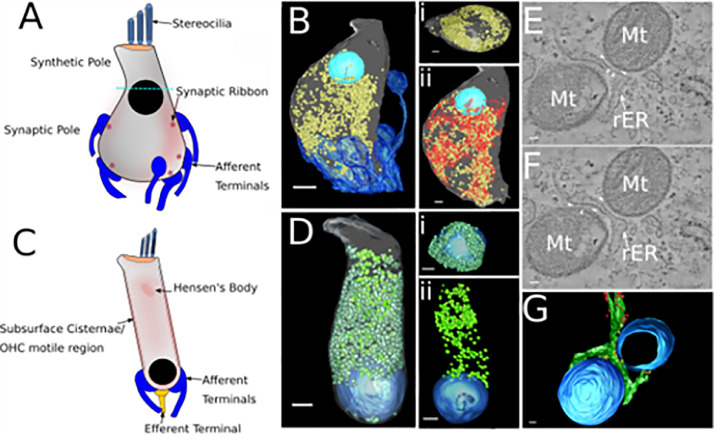
**Distribution of mitochondria in mouse hair cells visualised by SBEM.** A and C) Diagram of IHC (A) and OHC (C) showing areas of increased mitochondrial density (red shading). B) Reconstruction of SBEM imaging of mitochondria in the infranuclear ‘synaptic’ region of the IHC. Mitochondria (yellow), afferent terminals (dark blue) and nucleus (light blue) are shown. B i) Top view of the cell, showing mitochondria gathered around the cell membrane, with few mitochondria central in the cytosol. ii) mitochondria and rough endoplasmic membrane (red) are co-localised through most of the cell. D) Reconstruction of SBEM imaging in the OHC. Two populations of mitochondria are shown the mitochondria underlying the SSC at the periphery of the cell (dull green) and the mitochondria more centrally located in the cytosol (bright green). D i) Top view of the mitochondria underlying the SSC. ii) side view of centrally located mitochondria. All scale bars 2 μm. E and F) Electron tomography imaging of mitochondria (Mt) -rough ER (rER) tethers in mouse IHC, white arrowheads indicate tethers, images are a composite of 10 tomographic slices (approx. 5.5 nm in Z). G) Reconstruction of mitochondria-ER tethers from data in E&F. Mitochondria (blue), rough ER (green), membrane attached ribosomes (red) and tethers (yellow). All scale bars 50 nm. Images in B and E-G from data presented in (Bullen et al., 2015)***.*** Images in D author's own data, produced with the same methods as B.

Recent studies, using serial blockface electron microscopy (SBEM), provide evidence of how mitochondrial organization may support function in the infranuclear region of IHCs (Fig. 3). Mouse IHCs have more afferent terminals on their lateral (abneural) sides, which are densely populated by mitochondria. IHC mitochondria also preferentially colocalize with the largest membrane complexes (Bullen et al., 2015). Further work has classified mouse cochlear IHCs as “Type-A” or “Type-B”, which are tilted toward the abneural or neural axis respectively (Liu et al., 2021). Type-A IHCs have more mitochondria of larger volumes, especially in their basolateral region. However, ribbon volume and the volume of the closest mitochondrion do not appear to be correlated (Liu et al., 2021). The mitochondrial organisation in IHCs is likely specialised to support the energetic demands of fast and sustained vesicle cycling at the ribbon and local Ca^2+^ buffering at active synapses.

Mature mammalian OHCs have a distinct arrangement of mitochondria along the lateral cell membranes, a pattern which is established during development in parallel with their functional maturation (Weaver and Schweitzer, 1994) and can be seen along with an internal mitochondrial population by SBEM imaging and reconstruction (Fig. 2). Since the activity of prestin, the motor protein underlying OHC motility, is not directly dependant on ATP (Butan et al., 2022; Dallos et al., 2006; Zheng et al., 2000), the lateral mitochondria are not thought to satisfy energetic demands OHC motility directly. In proximity to prestin and the lateral membrane are the subsurface cisternae (SSC), a layered system of membranes surrounding the lateral circumference of the cytoplasm which has been implicated in cytosolic calcium homoeostasis and may also support OHC mechanics via its specialized internal structure (Triffo et al., 2019). The association of mitochondria with the SSC, rather than the HC lateral membrane (Perkins et al., 2020), may position them in the proximity of local Ca^2+^hot spots, allowing them to take up and transfer Ca^2+^ to PMCA1 pumps for extrusion. Mitochondria along the OHC lateral wall may therefore also regulate the activity of ion pumps and transporters that maintain ion homoeostasis throughout the cell.

## 4 Current views of mitochondrial ultrastructure in cells of the inner ear

### 4.1 Crista structure and orientation

Within individual mitochondria, the membranous cristae are fundamental structures that define the internal structure and function of a mitochondrion. Cristae are complex structures, the site of oxidative phosphorylation and mitochondrial ROS production. Individual cristae are functionally autonomous, capable of maintaining their own membrane potentials separate from others in the mitochondrion, allowing single cristae to perform distinct roles (Wolf et al., 2019). It has also been suggested that sensitivity of the lipid cardiolipin to Ca^2+^ enhances mitochondrial ATP production in excitable cells by modulating the mechanical properties of the inner mitochondrial membrane (Joubert and Puff, 2021). Mitochondrial cristae are also capable of dynamic reorganisation in response to cell state (Mannella, 2006). The balance between cristae and mitochondrial matrix volume affects mitochondrial function, with more cristae promoting greater energy production and larger matrix volume favouring biosynthetic and signalling functions (Cogliati et al., 2016; Glancy et al., 2020).

In addition to the number and structure of cristae in an individual mitochondrion, crista orientation also regulates interactions of mitochondria with each other, and with other organelles. At sites of inter-mitochondrial junctions, the crista orientation is coordinated between adjacent mitochondria, with the cristae forming parallel arrays consistent with electrochemical coupling (Picard et al., 2015). During inter-organelle communication between mitochondria and rough endoplasmic reticulum (RER), Ca^2+^ signal propagation from RER to mitochondria causes crista re-modelling characterised by crista compression, a concomitant increase in matrix volume, and alignment of the cristae junctions to the ER-mitochondrial interface. The latter are associated with increased levels of ROS-induced Ca^2+^ release (Booth et al., 2016).

### 4.2 Filamentous linkages

With the advent of high-resolution imaging, in particular electron tomography, the importance of filamentous linkages, or tethers, between mitochondria and endoplasmic reticulum has become increasingly apparent (Csordás et al., 2006) (Fig. 3E,F). Mitochondrial tethers to endoplasmic reticulum are important for communication between the two organelles, including the transfer of lipids and Ca^2+^ ions. Contacts tethering the two are maintained even as mitochondria and ER are moved along the cytoskeleton to different parts of the cell (Rowland and Voeltz, 2012). It has been shown that these contact sites are dynamic, with mitochondria and ER moving closer to each other during cellular stress and contacts reducing during nutrient oversupply. These filamentous linkages regulate numerous intracellular processes including redox signalling, inflammation and autophagy (Simmen and Herrera-Cruz, 2018).

Mitochondrial tethers to the RER have been observed in IHCs. Mitochondrial-ER linkages were observed in regions of the membrane concentrated around areas of the cell with the greatest number of afferent synapses. This suggests at least one function of these linkages is to facilitate buffering of Ca^2+^ in localised hot spots around the synapse (Bullen et al., 2015). In OHCs the lateral mitochondria are most strongly associated with the SSC. High-resolution imaging with electron tomography has revealed that lateral mitochondria are tethered to the SSC by filamentous links (Perkins et al., 2020). Moreover, under homoeostatic conditions, the cristae and crista junctions of these mitochondria are preferentially orientated toward the SSC. The close association of SSC, outer mitochondrial membrane and cristae junctions created by this arrangement is suspected to support efficient Ca^2+^ transport from the SSC to the mitochondrial matrix.

### 4.3 Ultrastructural features in HCs

The structure of cristae in HCs has been described as mostly lamellar in shape (Fig. 2A), indicative of mitochondria with high oxidative phosphorylation activity, perhaps unsurprising considering the metabolic requirements of HCs (Lesus et al., 2019). Outside the cochlea, studies in the calyx of Held in the brainstem have shown that the crista junctions of mitochondria in the active zones show orientation towards a cytoskeletal network that connects them with the presynaptic membrane. The majority of these crista junctions were evident on the side of the mitochondria facing the presynaptic membrane (Perkins et al., 2010). Preliminary work suggests that a similar polarisation of mitochondrial crista junctions is present in the apical regions of vestibular HCs and cochlear OHCs, with an orientation towards structures including stereociliary rootlets, the striated organelle, and Hensen bodies (Lesus et al., 2019).

## 5 Measuring mitochondrial structure, activity, metabolism and Ca2+ signalling in HCs

Cellular ATP is generated through both cytosolic glycolysis and OXPHOS in mitochondria. In glycolysis, phosphates are transferred from metabolic intermediates to ADP, producing its phosphorylated counterpart ATP. The tricarboxylic acid (TCA) cycle generates reduced nicotinamide adenine dinucleotide (NADH) and flavin adenine dinucleotide (FADH_2_). These electron donors supply electrons to the mitochondrial electron transfer chain (ETC), where they fuel ATP production at the ATP-synthase (ETC complex V). Glycolysis and the mitochondrial TCA cycle are linked at pyruvate, which is taken up into mitochondria via the mitochondrial pyruvate carrier. Overall, OXPHOS generates significantly more ATP than glycolysis.

To understand the functional role of mitochondria in HCs, and link this to their ultrastructural changes observed in during HC death, it is important to have robust methods for studying the activity of mitochondria in living cells of the normal auditory and vestibular systems. These have traditionally been difficult to implement in mammalian auditory tissues due to the cochlea's relative inaccessibility and the problems of maintaining HCs in culture. Discussed below are some of the methods that may be used to assay activity in the living system and what we understand of the role of mitochondrial metabolism in HC function.

Numerous methods have been implemented to examine cytosolic and mitochondrial metabolism in a variety of cochlear cell types in both fixed and live samples (see Table 1). Early work relied mainly on histochemical detection of mitochondrial enzyme activity. Although these methods revealed differences in mitochondrial structure and function, caution was required when interpretating these data due to the variability associated with different methods of sample preparation. More modern approaches have superseded classic histochemical techniques, permitting live imaging of mitochondrial activity and cellular biochemistry. Vital dyes sensitive to the mitochondrial membrane potential (ΔΨm) (Duchen et al., 2003; O'Sullivan et al., 2022), probes to monitor mitochondrial Ca^2+^ (Davidson and Duchen, 2012)***,*** fluorescence lifetime imaging (FLIM) of cellular NAD(P)H and FADH_2_ (Blacker et al., 2014; Jensen-Smith et al., 2012; Majumder et al., 2019; O'Sullivan et al., 2022), and genetically encoded sensors (Gökerküçük et al., 2020) are now available and have been used to study cellular metabolism and mitochondrial activity in cells of the auditory system (Esterberg et al., 2014; Manikandan et al., 2021; Mann et al., 2009; Pickett et al., 2018; Wong et al., 2019)***.***

**Table 1:** Methods for assessment of mitochondrial metabolism in vertebrate mechanosensory epithelia.

Method	Description	Readout	References
tetraethylbenzimidazolylcarbocyanine iodide (JC-1)	Fluorescent cationic dyes which localise to mitochondria	changes in fluorescence intensity (TMRM, TMRE, DASPEI) and wavelength (JC-1) correspond with changes in ΔΨm	(Pickett et al., 2018)
Tetramethyl Rhodamine ethyl ester (TMRE)			(Zholudeva et al., 2015)
2-{4-(dimethylamino)styryl}-N-ethylpyridinium iodide (DASPEI)			(Owens et al., 2007)
Tetramethyl Rhodamine methyl ester (TMRM)			(Shi et al., 2007; Desa et al., 2019; Perkins et al., 2020)
NADH/FADH_2_ fluorescence	NADH/FADH_2_ autofluorescence	Intensity of fluorescence corresponds with availability of reducing agents to fuel electron transport chain	(Tiede et al., 2007)
NAD(P)H Fluorescence lifetime imaging	NADPH autofluorescence is composed of multiple duration of enzyme bound vs. free species, reflecting the metabolic state of the cell	Ratio of NADPH/NADH acts as a metabolic "fingerprint"; higher NADPH is indicative of oxidative stress	(Desa et al., 2019; Majumder et al., 2019)
Mass spectrometry	Identification of metabolic enzymes in bulk tissue or cells	Presence and concentration of glycolytic/mitochondrial enzymes can be determined	(Spinelli et al., 2012)
ATP Assay	Measurement of ATP concentration through enzymatic means	Concentration of ATP reflects activity of the ATP synthase	(Chen et al., 2012a)
Electron tomography	Osmium tetroxide fixation and heavy metal staining allowing visualisation of mitochondrial structure	Detailed visualisation of cristae structure and crista junctions in 3D	(Perkins et al., 2020)
Electron microscopy		Mitochondrial size, crista morphology and matrix density can be assessed	(Spoendlin, 1971)
Immunoassay	Identification and quantification of proteins	Mitochondrial proteins can be identified and localised in the cell. Differences in fluorescence/chromogenic intensity can relate to changes in metabolism	(Lyu et al., 2020)
Transcriptomics	Identification and quantification of transcripts	Transcripts of mitochondrial proteins can be quantified and related to cell state changes. Levels of these transcripts may reflect metabolic differences, with the caveat that much regulation of metabolic proteins is carried out post-translationally.	(Baek et al., 2022)

## 6 The role of mitochondria and metabolism in hc function

**Fig. 4:**
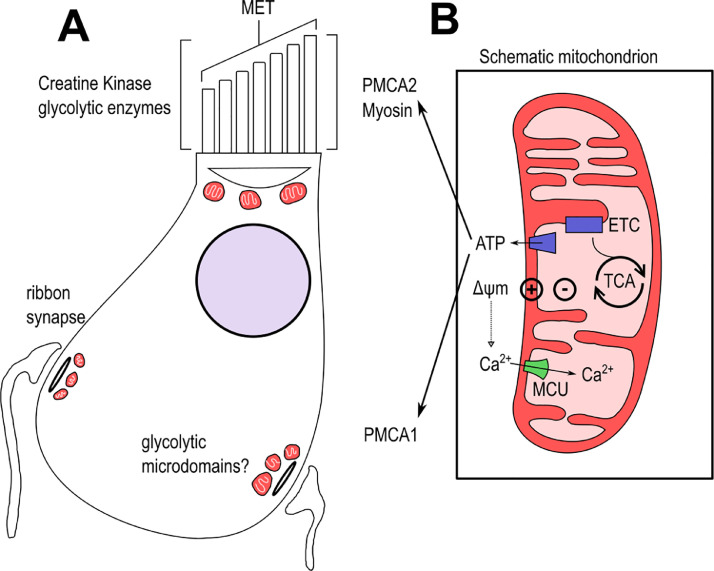
**Illustration of mitochondrial contributions to HC function.** A) diagram of a mammalian IHC. The stereocilia occupy the apical surface of the cell and are a centre of ATP use and calcium flux. Ca^2+^ enters the cell alongside *K*^+^ via the MET channels on the tips of the stereocilia. Mitochondria (red) beneath the cuticular plate are thought to supply ATP for Ca^2+^ clearance via PMCA2, as well as myosin adaptation. However, creatine kinase and glycolytic enzymes in the stereocilia also provide ATP for these processes. Mitochondria are also required for Ca^2+^ buffering in proximity to the basolateral ribbon synapses. Ca^2+^clearance from this region of the cell is via PMCA1, which also requires ATP. Glycolytic microdomains of the cytoplasm may also provide ATP in these regions. B) Schematic of a generic mitochondrion showing the relevant pathways. ATP is produced by the electron transport chain (ETC) fuelled by the TCA cycle. The generation of a proton gradient by the ETC is responsible for the formation of the mitochondrial membrane potential (ΔΨm). The negative charge produced within the mitochondrial matrix is critical of regulating Ca^2+^ import during buffering via the mitochondrial calcium uniporter (MCU).

Mitochondrial function is multifaceted. Mitochondria provide a structural housing for the TCA cycle and oxidative phosphorylation (OXPHOS) the combined activities of which generate ATP and metabolic intermediates alongside regulating cellular redox state. In addition, they are mediators of apoptosis (Wang and Youle, 2009), are a major source of ROS (Sies and Jones, 2020) and regulate cytosolic Ca^2+^signalling (Giorgi et al., 2018; Mammucari et al., 2018). As discussed above, the specific aspects of HC function, each with its own set of metabolic demands, are compartmentalised into different regions of the cell. Mitochondria play a central role in regulating Ca^2+^ homoeostasis in the different HC compartments during transduction and synaptic transmission. Large numbers of mitochondria are concentrated just beneath the cuticular plate of the HC, presumably to supply the ATP needed to facilitate the activity of myosin motors during adaptation and sustain the extrusion of Ca^2+^via plasma membrane calcium ATPase2 (PMCA2) transporters during transduction (Fig. 4) (Shin et al., 2007). Synaptic transmission also requires ATP to facilitate vesicle priming and neurotransmitter uptake (Pathak et al., 2015). Synapses in the basolateral potion of HCs are therefore associated with high numbers of presynaptic mitochondria (Spicer et al., 1999, 2007)***.***

### 6.1 A role for mitochondria in mechanosensory transduction

As mentioned above, mechanotransduction is the process by which mechanical sound stimuli are converted into electrical activity in HCs. Sound-induced deflections of the HC stereociliary bundle activate mechanoelectrical transduction (MET) channels located at their tips. This elicits subsequent firing in auditory nerve fibres. The MET channel functions as a non-selective cation channel, with the highest permeability being to Ca^2+^ and roughly equal permeabilities to Na^+^ and *K*^+^. Due to the composition of the cochlear endolymph, a high *K*^+^solution that bathes the apical portion of the HC, the MET current is carried predominantly by *K*^+^ (Fettiplace, 2017; Fettiplace and Kim, 2014). Although ATP is not required for transduction itself, it does play an important role in regulating both adaptation and force generation in the HC bundle. It has been suggested that sufficient levels of ATP are essential for maintaining the optimal sensitivity of transduction.

### 6.2 Mitochondrial Ca^2±^handling in HCs

In a vast majority of cell types, mitochondria accumulate Ca^2+^during all increases in cytosolic Ca^2+^. The amount of Ca^2+^ sequestered is determined by the amplitude and duration of the Ca^2+^signal in addition to its source. A significant amount (∼10%) of the MET channel current is carried by Ca^2+^ originating from the cochlear endolymph (Beurg et al., 2009, 2006; Ricci et al., 2003)***.*** The majority of this Ca^2+^ is cleared by rapid transfer to mobile Ca^2+^ buffers such as Calbindin D28K, the PMCA pumps and to mitochondria. When cytosolic Ca^2+^ increases to a certain level during transduction, mitochondria function as fixed buffers and sequester Ca^2+^ via the mitochondrial Ca^2+^ uniporter (MCU) allowing the optimum Ca^2+^ level for MET channel function to be maintained (Beurg et al., 2010; Mammucari et al., 2018; Rizzuto et al., 2012).

HC function relies on active mitochondria because Ca^2+^uptake depends on the mitochondrial membrane potential, an electrochemical potential gradient of roughly −180 mV between the cytosol and mitochondrial matrix (Rottenberg and Scarpa, 1974; Vasington and Murphy, 1962). Mitochondrial Ca^2+^ uptake follows a sigmoidal response characterised by low uptake at resting cytosolic Ca^2+^ levels and high handling capacity in response to large cytosolic Ca^2+^ loads. The sigmoidal nature of MCU-dependant Ca^2+^ uptake coupled with the specific positioning of mitochondria throughout the HC ensures they can respond rapidly to local changes in cytosolic Ca^2+^ during stimulation. As hypothesised from studies in other cell types, given the low Ca^2+^ affinity of the MCU, Ca^2+^ levels in stereocilia and at the cuticular plate would need to increase well above 1 mM before significant uptake and buffering is observed (De Stefani et al., 2016)***.*** The cuticular plate prevents mitochondria from contacting the bases of stereocilia.

The high concentration of mitochondria in this region may therefore act as a firewall to constrain Ca^2+^ that enters stereocilia during transduction. It has been suggested that mitochondria are present in the cuticular plate-free region, but the functional and differences between these two apical populations remain at present unknown. Higher resolution imaging studies are needed to discern the specific dynamics of Ca^2+^ buffering in the different mitochondrial populations at the apical HC membrane.

### 6.3 Mitochondria in hc adaptation

Following MET channel opening, HCs adapt to maintain their sensitivity to stimulation. Adaptation consists of two independent processes operating on fast and slow timescales, both of which are highly regulated by intracellular Ca^2+^. During transduction it is therefore vital that stereociliary Ca^2+^ be tightly controlled and maintained within a physiological range (Fettiplace, 2017; Fettiplace and Kim, 2014; Mammano, 2011)***.*** The rate and extent of adaptation is determined by the Ca^2+^ influx through the MET channel and the Ca^2+^ buffering capacity by mobile and fixed buffers within stereocilia and at the cuticular plate (Corns et al., 2014). If Ca^2+^levels in stereocilia become too low, this significantly alters the open probability of the MET channel at rest (Beurg et al., 2008; Peng et al., 2013; Ricci et al., 1998; Ricci and Fettiplace, 1997; Shin et al., 2007). If Ca^2+^ levels become too high, this probably leads to cytosolic Ca^2+^ overload and cell death (Rizzuto et al., 2012). In guinea pig OHCs mitochondria are seen concentrated beneath the cuticular plate in close association with the Hensen body, where they are thought to buffer Ca^2+^ that has entered through the MET channel during transduction (Corns et al., 2014; reviewed in Mammano, 2011)***.*** The different modes of Ca^2+^regulation and buffering by mitochondria between stereocilia in the bundle and the HC body can be likened to that seen in a dendrite between its spine and body (Yuste et al., 2000) or to that described in pancreatic acinar cells during insulin secretion (Petersen and Tepikin, 2008)***.***

A third adaptation mechanism has also been described in HCs of the turtle papilla, where the MET channel activation range is regulated metabolically by cAMP (Ricci and Fettiplace, 1997)***.*** The extensive crosstalk between cellular ATP, cytosolic Ca^2+^and cAMP (Di Benedetto et al., 2014) and the fact that they all converge at the mitochondrion, highlights a central role for this organelle in providing sufficient ATP during mechanotransduction. Mitochondrially-derived ATP is also likely important for sustaining the activity of the motor protein Myosin1c during slow adaptation (Gillespie and Cyr, 2004; Phillips et al., 2006). The specific mechanisms linking these three signalling networks in HCs remain elusive and require further investigation. Additionally, as the mechanisms of transduction show subtle differences between HC subtypes and across species, the specific modes of mitochondrial Ca^2+^buffering and mitochondrial activity across different models may be context dependant.

### 6.4 Metabolic regulation of hc pmca pumps

PMCA activity accounts for the majority of Ca^2+^ extrusion in stereocilia under physiological conditions, with mitochondria sequestering only small amounts (Beurg et al., 2010; Fettiplace and Nam, 2019). Plasma membrane Ca^2+^-ATPase (PMCA) isoforms are cell-specific, show distinct modes of regulation and display unique biochemical properties (Bruce, 2018). HCs express the isoforms PMCA1 and PMCA2a, whose location is segregated into different cellular compartments (Dumont et al., 2001). PMCA1 is expressed in the basolateral membranes of HCs and glial-like SCs which reside as a mosaic with HCs in inner ear sensory epithelia. PMCA2a is expressed exclusively in the stereociliary bundle, where it is the primary mode of Ca^2+^ clearance following MET channel activation (Q. Chen et al., 2012; Dumont et al., 2001). The activity of these pumps is regulated by both Ca^2+^ proteolysis and the availability of ATP.

Sustained active pumping of HC PMCAs is essential for maintaining physiological Ca^2+^ levels within stereocilia and in the HC body. This sustained activity requires a constant and rapid supply of ATP. The plentiful mitochondria and metabolic enzymes throughout the HC body and concentrated at the basolateral membrane (Bullen et al., 2015; Dumont et al., 2001; Kolla et al., 2020; O'Sullivan et al., 2022) probably generate sufficient levels of ATP to maintain activity of PMCA1. ATP production in stereocilia however is more complicated as these structures are devoid of mitochondria. Although some ATP can diffuse towards stereociliary tips from cuticular plate mitochondria located at their bases, this is neither sufficient nor fast enough to support the high PMCA2a activity required for transduction (Shin et al., 2007). Analysis of protein expression in stereocilia using mass-spectrometry has revealed high levels of glycolytic enzymes and expression of the brain-specific isoform of creatine kinase (bCK). Further experimentation showed that to carry out the demanding task of Ca^2+^ homoeostasis during transduction, HC stereocilia activate bCK and consume ATP generated locally through glycolysis (Shin et al., 2007). A similar phenomenon has been described in skeletal muscle, where a phosphocreatine (PCr) shuttle system (Bessman and Geiger, 1981) facilitates the subcellular distribution of high energy phosphates throughout the cell.

A model of Ca^2+^ homoeostasis has been proposed in transducing OHCs, whereby Ca^2+^is bound initially by mobile calcium binding proteins and then unloaded to PMCA2a. It is only during larger Ca^2+^ loads, that mitochondrial buffering plays a significant role (Fettiplace and Nam, 2019). As the MET channel conductance increases significantly in amplitude from high-to-low frequency regions (Beurg et al., 2006; Fettiplace, 2017), the Ca^2+^ buffering network in high frequency HCs must be primed to handle larger Ca^2+^ loads. It is plausible therefore that mitochondria have a more significant role in cytosolic Ca^2+^ homoeostasis in high-frequency HCs of the basal/proximal cochlea and may explain their increased vulnerability to impaired mitochondrial function.

### 6.5 Mitochondrial function in the hc body and at the synapse

Mitochondria play an important role in clearing Ca^2+^ from the presynaptic terminal during transmission in excitable cells. Presynaptic mitochondria either sequester Ca^2+^ directly, or fuel Ca^2+^ removal through secondary mechanisms such as PMCA pumps and the Na^+^-Ca^2+^ exchanger (Johnson et al., 2007). PMCA-dependant Ca^2+^ clearance has recently been identified as an important mode of Ca^2+^ buffering at the HC ribbon synapse in the bullfrog papilla (Cuadra et al., 2021). Given the similar dynamics observed in presynaptic Ca^2+^ signals measured in HCs from mice (Bortolozzi et al., 2008; Frank et al., 2009), frogs (Rispoli et al., 2001) and turtles (Tucker and Fettiplace, 1995) during transmission, this buffering mechanism may be present at HC ribbon synapses across species. In retinal bipolar cells mitochondria function primarily as an energy source to fuel Ca^2+^ extrusion through PMCAs (Zenisek and Matthews, 2000). Similar to the Ca^2+^ buffering observed in the apical part of the HC during transduction, mitochondria in bipolar cells only sequester Ca^2+^ in the absence of PMCA activity, or if cytosolic levels increase above a certain set point (Zenisek and Matthews, 2000). Mitochondria have also been shown to regulate neurotransmitter uptake and vesicle priming but not transmitter release and vesicle fusion in retinal bipolar neurons (Heidelberger et al., 2002). The specific modes of mitochondrial Ca^2+^ uptake under different physiological conditions and the mitochondrial regulation of vesicle priming and fusion have not been explored in detail at the HC ribbon synapse.

There is evidence supporting a role for glycolytic microdomains in the presynaptic terminals of central synaptic ribbons. Biochemical analysis of synaptosomes and synaptic vesicles from presynaptic compartments has shown high expression of glycolytic enzymes involved in the ATP-yielding steps of glycolysis (Ikemoto et al., 2003). Generation of glycolytic ATP in presynaptic microdomains is also thought to regulate the molecular events associated with vesicle fusion and membrane remodelling (Rangaraju et al., 2014)***.*** Additionally, in C. elegans, glycolytic metabolism was identified as the major source of ATP that supports sustained synaptic function during periods of stress (Jang et al., 2016)***.*** The role of glycolysis has not been explored at the HC ribbon synapse under either physiological or pathological conditions. Tandem mass-spectrometry in HCs from the chick basilar papilla have however identified high levels of glycolytic enzymes associated with the same ATP-yielding steps in glycolysis. This analysis also revealed subtle differences between the HC ribbon and those in other cell types, when comparing the expression levels of enzymes involved in the TCA-cycle and mitochondrial oxidative phosphorylation (Uthaiah and Hudspeth, 2010). The unique metabolic signatures at HC ribbon synapses could be associated with the specific metabolic requirements necessary for encoding complex sound stimuli. The role of different metabolic pathways in regulating synaptic transmission in HCs or at different frequency positions along the cochlea have not been explored.

Nicotinamide adenine dinucleotide (NAD) and its phosphorylated counterpart nicotinamide adenine dinucleotide phosphate (NADP) are involved in most cellular redox reactions as the shuttle electrons between the many metabolic pathways in a cell. A role for mitochondria and cellular redox state was recently identified in regulating both the development and function of HC ribbon synapses in zebrafish HCs. Live imaging of HCs from zebrafish neuromasts showed mitochondrial Ca^2+^ uptake following Ca^2+^ influx through Ca_v_1.3 channels during synaptic transmission. Inhibiting this uptake significantly impacted the size and integrity of synapses, revealing a role for mitochondria in the development and long-term function of the ribbon synapse (Wong et al., 2019). This regulatory mechanism is thought to be dependant on the cellular redox state resulting from the NAD^+^/NADH ratio. Alterations in the size of ribbon synapses have also been reported in aged C57Blk6 mice, where the cellular redox state and NAD^+^/NADH balance is dysregulated (Peineau et al., 2021)***.*** The ribbon itself is constructed through interactions between adjoining Ribeye protein monomers. As these interdomain interactions are regulated by the availability of cellular NAD^+^, changes in the metabolic state of a HC would likely affect the structure and function of the ribbon synapse.

## 7 Mitochondria in hc damage and death

Mitochondrial dysfunction is a hallmark in a range of pathologies including the main types of hearing loss (ototoxic drug-induced, noise-induced, and age-related). Here, mitochondrial dysfunction refers to impaired activity in the mitochondrial ETC, collapse of the mitochondrial membrane potential (ΔΨm) or activation of the permeability transition pore (PTP) during HC death. In a majority of cases this occurs due to cytosolic Ca^2+^ dysregulation and increased levels of cellular reactive oxygen species (ROS). These changes in cell signalling and mitochondrial activity are also seen in a number of mitochondrial syndromes that present with associated hearing loss (Gorman et al., 2016). For a more detailed review of work investigating mitochondrial mutations in syndromic and non-syndromic deafness, we refer the reader to other literature (Huth et al., 2011; Kamogashira et al., 2015; Liu et al., 2010). It is important to note that certain mitochondrial mutations do cause increased susceptibility to hearing loss and that they affect HCs in the cochlea more than those in vestibular organs (Huth et al., 2011). These differences may reflect distinct metabolic demands and metabolic states between the two systems. It is also plausible that mutations causing mitochondrial impairments have a greater impact on cells with higher metabolic demands. For example, high versus low frequency HCs in the cochlea and type I verses type II HCs in the vestibular system (Huth et al., 2011). Mitochondrial mutations and diseases also significantly impact cells in the stria vascularis (Suga and Lindsay, 1976). This section will discuss what we currently understand about the response of otherwise normal mitochondria to ototoxic insult and damaging noise stimuli, and how changes in mitochondrial metabolism, Ca^2+^ homoeostasis and ROS generation during the latter contribute to HC death.

### 7.1 Changes in mitochondrial activity during ototoxic and noise-induced damage

Aside from their role in metabolism, mitochondria are key regulators of apoptosis and necrosis (Wang and Youle, 2009)***.*** A vast majority of pathways leading to cell death are associated with increased levels of cellular reactive oxygen species (ROS), which in the context of ototoxicity and noise damage has long been considered a biproduct of dysregulated metabolism (Kamogashira et al., 2015)***.*** Non-canonical cell death mechanisms are often evoked by increased ROS production, cytosolic Ca^2+^ dysregulation and reduced cellular ATP (Bauer and Murphy, 2020; Hajnóczky et al., 2006; Rasola and Bernardi, 2011). The coincidence of the latter causes mitochondrial PTP activation and ultimately cell death via necrosis. This pathway has been documented in various models of HC cell death and will be discussed below.

### 7.2 Mitochondrial bioenergetics, Ca^2±^ signalling and ros during drug-induced ototoxicity

Aminoglycoside antibiotics (AAs), used world-wide in the treatment of gram-negative bacterial infections, pose a major challenge to HCs of the cochlear and vestibular organs (Ariano et al., 2008; Black et al., 2004; Forge and Schacht, 2000; Huth et al., 2011; Jiang et al., 2017). AAs enter through the HC MET channels (Alharazneh et al., 2011; Bird et al., 2010; Marcotti et al., 2005; Rizzi and Hirose, 2007) where they generate large amounts of ROS (Clerici et al., 1996; Hirose et al., 1997) and accumulate in mitochondria, lysosomes, Golgi bodies and ER (Steyger et al., 2003). It is likely that the combination of aminoglycoside accumulation coupled with the high metabolic demands of HCs renders them highly vulnerable to these drugs. There are numerous mitochondrial mutations that cause increased sensitivity to AAs in patients (Gao et al., 2017; Nguyen and Jeyakumar, 2019)*.* The ototoxic and vestibulotoxic effects vary across the AA family, with some showing higher potency in one organ over the other. Differences in the choice of AA and the specific treatment regime used underly the variations in the incidence of HC death reported across studies in animal models (Huth et al., 2011). Of particular interest here, are the differences observed in the timing of AA action across species and HC subtypes in vitro*,* when using explant culture models of auditory and vestibular epithelia (Bird et al., 2010; Dehne et al., 2002; Esterberg et al., 2013; Goodyear et al., 2008; Jagger et al., 2014; Lahne and Gale, 2010, 2008; Mann et al., 2009; O'Reilly et al., 2019). More specifically, these studies show that Ca^2+^dysregulation, loss of mitochondrial integrity and ROS generation vary between species and across HC subtypes during the acute phase of AA toxicity.

### 7.3 A role for mitochondria and cell metabolism in noise damage

Exposure to intense sound stimuli causes temporary or permanent threshold shifts alongside altered activity in auditory nerve fibres (Kurabi et al., 2017). Significant noise causes mechanical disruption of the cochlear sensory epithelium, which in extreme cases breaks the barrier between endolymph and perilymph, causing *K*^+^ dysregulation throughout the HC. More commonly noise damage is characterised by biochemical changes within the cell itself (Kurabi et al., 2017; Liberman and Beil, 1979; Slepecky, 1986)***.*** Noise-induced HC damage and drug-induced ototoxicity share common hallmarks. Primarily, acute metabolic overstimulation is thought the major prerequisite to HC death in both scenarios (Huth et al., 2011; Kamogashira et al., 2015; Kurabi et al., 2017). Furthermore, as with ototoxicity, increased cellular ROS (Henderson et al., 2006; Liu et al., 2010; Vlajkovic et al., 2010) elevated cytosolic Ca^2+^ levels (Fridberger et al., 1998; Wang et al., 2019) and impaired mitochondrial function (Bischoff et al., 1970; Chen et al., 2022; Ji et al., 2019; Park et al., 2012; Spoendlin, 1971) have been reported in noise-damaged HCs.

Oxidative stress can be defined as the inadequate clearance or excessive production of ROS (Sies and Jones, 2020). The ability of a cell to counteract oxidative stress within the ever-changing environment of its niche is essential for survival. One way metabolism protects cells against oxidative stress is through the cellular antioxidant machinery that controls levels of the reducing coenzyme NADP. Reduced NADP (NADPH) is generated through the oxidative branch of the pentose phosphate pathway (PPP), providing the largest source of antioxidant defence in the cytosol (Moon et al., 2020) and in mitochondria through the activity of NADPH-producing enzymes (Dey et al., 2016)***.*** Studies using the antioxidants d-methionine (Cheng et al., 2008) and mitoTEMPO (Chen et al., 2022) have shown protection against noise-induced oxidative stress. In other cell types, methionine is known to stimulate PPP activity leading to increased levels of cellular NADPH (Campbell et al., 2016). Metabolomics analysis of inner ear tissue have shown that methionine levels, increase following noise trauma (Ji et al., 2019)***.*** In the cochlea***,*** d-methionine may therefore exert its antioxidant effects by stimulating activity in the oxidative branch of the PPP and increasing the availability of cytosolic NADPH. It is not presently clear how noise trauma affects the expression levels of NADPH-generating enzymes in HC mitochondria. Although NAD(P)H metabolism has been studied during AA-induced ototoxicity, changes in PPP activity or in the redox state of cytosolic and mitochondrial NADPH pools have not been explored in either ototoxicity or noise-induced HC damage. Understanding how the HC antioxidant and metabolic networks are regulated during noise and ototoxic insults highlights a promising avenue for future investigations.

### 7.4 Changes in mitochondrial ultrastructure during hc degeneration and damage

As discussed, mitochondrial ultrastructure and distribution appear to be highly regulated to support HC function, and it is known that acute and chronic stress of auditory cells induces disruption of the mitochondrial inner membrane architecture. Mitochondrial-SSC decoupling has been reported in OHCs during ageing and noise trauma is known to elicit generalised mitochondrial damage in rodents (Bischoff et al., 1970; Spoendlin, 1971). More recent investigations using 3D electron tomography in the aged mouse cochlea provide a clearer picture of how mitochondrial morphology and distribution are disrupted. Ageing leads to a loss of mitochondrial crista integrity in both IHCs and OHCs of C57BL/6 mice (Lyu et al., 2020; Perkins et al., 2020). In aged HCs the organisation of mitochondria becomes disrupted and the overall number of mitochondria within the cell is reduced (Perkins et al., 2020). A primary role proposed for SSC-mitochondrial connections is coupling the apical and basal parts of the HC. Decoupling of these networks, as observed in OHCs, was associated with fusion/fission imbalances, Ca^2+^ dysregulation and energetic stress (Perkins et al., 2020)***.*** In addition to changes in mitochondrial crista morphology, mitochondrial swelling and changes in the mitochondrial matrix composition have been observed in HCs from aged mice and neomycin-exposed zebrafish (Lyu et al., 2020; Owens et al., 2007; Perkins et al., 2020). The specific mechanisms regulating the morphological changes observed in mitochondria during HC death and damage remain at present unclear.

Having established that changes in mitochondrial morphology and ultrastructure accompany HC death it is important to understand how these are reflected in the mitochondrial activity of compromised HCs. Using the potentiometric dyes TMRM and Rhodamine 123 to monitor the mitochondrial membrane potential (ΔΨm) and high resolution respirometry techniques to measure respiration, changes in mitochondrial activity have been investigated during HC damage and ototoxicity (Desa et al., 2019; O'Reilly et al., 2019; Owens et al., 2007; Shi et al., 2007; Zholudeva et al., 2015). Studies in isolated mitochondria have also shown that AAs directly impact mitochondrial ETC activity leading to a collapse of ΔΨm (O'Reilly et al., 2019). AA-induced changes in ΔΨm have been studied in combination with cellular NAD(P)H biochemistry and mitochondrial ROS production in cells of the murine cochlea (O'Reilly et al., 2019). Here, acute ototoxic insult evokes an immediate yet transitory hyperpolarisation of ΔΨm followed shortly by its collapse. Impaired mitochondrial activity was accompanied by changes in cellular NAD(P)H biochemistry and increased generation of mitochondrial ROS was observed using the live dye Mitosox Red. Taken together, these data indicate a significant reduction in mitochondrial complex II activity during AA toxicity (Desa et al., 2019; Zholudeva et al., 2015)***.*** This work also showed that AAs evoked both cell and frequency-specific changes in NAD(P)H metabolism which plays a major role in regulating cellular antioxidant defence during stress (Zholudeva et al., 2015)***.***

Changes in cellular ATP have been observed during HC degeneration in response to noise (Chen et al., 2012a; Chen et al., 2022) and during gentamicin toxicity (Tian et al., 2020). Depleting ATP levels in OHCs, and reducing the availability of pyruvate to fuel the TCA cycle and mitochondrial OXPHOS in UB/OC1 cells, an inner ear cell line, increased the extent of cell death observed after ototoxic insult (Chen et al., 2012a; Tian et al., 2020). These findings highlight transient “energetic depletion” as a major prerequisite for HC death.

### 7.5 Mitochondrial Ca^2^^+^dysregulation and activation of the PTP during ototoxicity

The most extensively studied cellular functions associated with mitochondrial Ca^2+^handling are localised and general Ca^2+^ buffering and the execution of cell death. The conclusions drawn from this multitude of studies differ depending on the specific cell models and pharmacological interventions used. For a detailed review of this literature, the reader is referred to previous articles (De Stefani et al., 2016). Under normal physiological conditions, uptake of Ca^2+^into mitochondria activates the rate limiting enzymes pyruvate dehydrogenase, α-ketoglutarate and isocitrate dehydrogenase, all of which are associated with the transfer of electrons to complex I in the mitochondrial ETC. In most cases activation of these enzymes leads to an increase in cellular ATP (McCormack et al., 1990; Rutter and Denton, 1988). However, under certain conditions Ca^2+^ signal propagation into mitochondria instead activates the PTP, a large conductance channel located in the inner mitochondrial membrane permeable to solutes up to 1 kDa. In many cases PTP activation is coincident with increased cellular ROS and prolonged elevations in cytosolic Ca^2+^ (Hajnóczky et al., 2006; Rasola and Bernardi, 2011) both of which are observed during HC death (Esterberg et al., 2016, 2014, 2013; Gale et al., 2004; Goodyear et al., 2008; Mann et al., 2009; Matsui et al., 2004; Steyger, 2021; Warchol, 2010). Extended periods of mitochondrial Ca^2+^ loading also produce ROS and in addition, significant amounts of free fatty acids. The combined effect of these two events is a sustained activation of the PTP, mitochondrial swelling and eventual rupture of the mitochondrial outer membrane leading to activation of cell death via apoptosis or necrosis.

Using genetic reporters to monitor cytosolic and mitochondrial dynamics in zebrafish during AA exposure, it has been possible to decode the signalling between mitochondrial, ER and the cytosolic compartments during HC death (Esterberg et al., 2016, 2014, 2013; Lee et al., 2022; Pickett et al., 2018). Like AA-induced signalling in mammalian HCs, exposure of lateral line HCs to neomycin or cisplatin causes rapid collapse of ΔΨm, followed shortly by elevations in cytosolic Ca^2+^. Blocking these Ca^2+^ transients using either pharmacological inhibitors or uncaging of Ca^2+^ chelators significantly mitigated the extent of AA toxicity (Esterberg et al., 2013). It was subsequently found that transfer of Ca^2+^between mitochondria and ER during ototoxicity plays an important role in the regulation of both cellular bioenergetics and intercellular Ca^2+^ homoeostasis. Under physiological conditions zebrafish HC mitochondria efficiently buffer small Ca^2+^ loads following its release from the ER during stimulation (Esterberg et al., 2014). This in turn causes hyperpolarisation of ΔΨm and stimulates mitochondrial activity (Brookes et al., 2004)***.*** Transient mitochondrial depolarisations caused by opening of the PTP, through a process known a flickering, (Agarwal et al., 2017; Ben-Hail and Shoshan-Barmatz, 2014; Duchen et al., 1998; MacVicar and Ko, 2017) drive Ca^2+^ efflux into the cytosol and act to re-set mitochondrial Ca^2+^ to physiological levels. Dysregulation of the Ca^2+^ transfer between mitochondrial and ER compartments is thought to underlie AA-induced toxicity in zebrafish HCs (Esterberg et al., 2014)***.*** In many other systems including mammalian HCs, inhibition of mitochondrial PTP activation protects against cell death and excitotoxicity (Baines et al., 2005; Dehne et al., 2002; Kroemer et al., 2007). In contrast to these models, inhibition of mitochondrial PTP in lateral line HCs renders them more susceptible to damage following AA exposure.

Studies in zebrafish lateral line HCs have shown that mitochondrial Ca^2+^ uptake underlies the generation of toxic levels of ROS during AA exposure. Blocking this Ca^2+^ uptake using Ru360, an inhibitor of the mitochondrial Ca^2+^ uniporter, reduced the levels of ROS generated in both the mitochondria and the cytosol (Esterberg et al., 2016)***.*** Drugs or small molecules that target mitochondrial ROS production are therefore promising therapies to ameliorate AA-induced ototoxicity. The role of mitochondrial Ca^2+^uptake is less clear in mammalian HCs. Here, Ru360 delays the onset of AA-induced HC death but does not provide long-term protection. The subtle differences in signalling between zebrafish and mammalian HCs during the acute phase of AA exposure indicate species-specific differences in the mechanisms underlying ototoxicity and HC death. With the improvement of imaging techniques allowing cellular events to be observed with greater precision and resolution, the signalling mechanisms underlying ototoxicity will likely become ever clearer in coming years.

## 8 Regulation of cellular metabolic flux and transcription

The metabolic flux in a cell is subject to constant modulation through allosteric modification of metabolic enzymes or following changes in the transcriptional regulation of metabolic genes (Bulcha et al., 2019)***.*** Flux through metabolic networks is highly regulated at multiple levels to maintain cellular homoeostasis in an ever-changing external environment. For example, in cancer cells, the metabolic network becomes re-wired to support accelerated proliferation and biosynthesis (Warburg, 1956; DeBerardinis and Thompson, 2012)***.*** Despite a significant knowledge of how metabolic genes are regulated, we understand less about how such transcriptional changes are reflected at the level of the metabolic network and energy output and the impact of these changes on cell physiology. Such analysis will be necessary if we are to understand how reprogramming of flux through the metabolic network impacts physiological function in different inner ear cell types.

Elegant work in *C. elegans,* combining transcriptomics with metabolic pathway analysis identified several transcription factors within a gene regulatory network (GRN) whose promoter activity was upregulated following perturbations in mitochondrial ETC activity (Bhattacharya et al., 2022). This work provided great insight into how changes in mitochondrial function can impact transcription factor activity within a GRN and thus the expression of target genes in a given cell. Understanding how metabolic flux can impact cell fate and function in a multicellular tissue such as the cochlea relies on the challenging task of integrating large transcriptional, proteomic and metabolomic data sets across developmental stages, cell types and spatial location. Despite significant advances in protocols for multi-omics analysis, their application in the inner ear remains technically challenging and requires significant optimisation. Integrating these complex data sets at the single cell level in inner ear sensory patches has great impact for what we understand about how metabolic reprogramming influences gene expression and cell fate decisions during development, regeneration and damage. Such analyses could help identify novel molecular mechanisms underlying congenital auditory defects and aid in developing therapeutic strategies to protect against HC death.

### 8.1 Spatial analysis of the transcriptome and metabolome

The correct function of inner ear structures relies on the precise spatial arrangement and patterning on its cells. Classical techniques for used for transcriptional and metabolic analysis often involve the destructive extraction of RNA, protein or metabolites using bulk assays on cell populations. Although advances in single cell technology allow the transcriptomic, epigenetic, and metabolic state of a cell to be studied, application of these techniques in the inner ear relies on the mechanical or enzymatic disruption of cells. As a consequence, important information regarding the spatial patterns of gene expression and metabolic activity are lost. Without positional information, it is difficult to determine how transcriptional and metabolic networks are regulated across the different inner ear structures and cell types. Over the past decade, there have been significant advances in high-throughput technologies to study gene expression spatially within tissues. Detailed description of these techniques goes far beyond the scope of this article, and we therefore refer the reader to recent reviews outlining major advances in spatial transcriptomics, metabolomics, and mass spectrometry (Alexandrov, 2020; Moses and Pachter, 2022). Optimising spatially resolved omics for different inner ear cell types will significantly further what we understand about the complex biology underlying their development, functional refinement and degeneration.

## 9 Summary and conclusion

Mitochondria are heterogeneous both across cell types and within single cells. Over recent years, it has become ever clearer that mitochondrial morphology, their network architecture and their subcellular localisation have important implications for cell physiology and cell death. The variation in shape, ultrastructure and physiology of HC mitochondria suggest that they undertake multiple roles in HCs. As discussed, these roles are location specific and are determined by the unique morphologies of mitochondria in the different populations. The links documented between mitochondria and ER in HCs suggest that to generate a deeper understanding of HC function, mitochondria should perhaps be considered in the context of their functional relationships with other organelles in the cell rather than as separate entities. This is especially relevant in HCs, given the importance of Ca^2+^ in mechanotransduction, synaptic transmission and cell death. Understanding the role and mechanisms of mitochondrial-ER Ca^2+^in mammalian HCs has significant impact for developing therapeutic strategies to protect against ototoxicity.

As revealed from studies in stem and cancer cell biology instructive signals from the surrounding niche also play an important role in regulating cell metabolic state, redox homoeostasis, and mitochondrial function. It will therefore be important to understand the reciprocity between HCs, their surrounding niche at different positions along the cochlea and their mitochondrial populations. With this knowledge the signalling between mitochondria, other organelles and their surrounding environment can be interrogated experimentally in functional and damaged HCs.

Understanding the functional role of different mitochondrial populations in HCs and how they are coupled to HC physiology, requires a multi-scale view of the mitochondrial network throughout the cell. It is therefore important we understand how the architecture of mitochondrial networks in HCs develops and which molecular mechanisms regulates it. This would include investigating how mitochondrial morphology and function are refined during HC maturation, as well as understanding the mechanisms involved in trafficking and positioning of mitochondria to different HC compartments. Very little is known about differences in mitochondrial physiology and metabolic function between different types of HC, for instance high and low-frequency HCs along the cochlea or type 1 and type 2 hair cells in the vestibular system. With a better understanding of mitochondrial morphology, network architecture and how this correlates with their metabolic role in cells of the normal cochlea, we can investigate how these might be altered in congenital hearing defects and during HC death. Our recent work investigating metabolism in the developing chick basilar papilla has also revealed an important role for cytosolic glycolysis and cellular NADPH/NADH in tonotopic patterning (O'Sullivan et al., 2022). Further studies investigating cross talk between developing mitochondria and metabolism in the local niche within the HC will have significant impact for what we understand about HC formation and functional refinement.

Through correlating high-resolution imaging of biochemical processes within individual mitochondria, with three-dimensional quantification of their morphology, it will be possible to discern more clearly the specific roles of mitochondria within functionally distinct HC compartments during homoeostasis and disease. With a clear understanding of the relationships between mitochondrial structure, distribution and activity in different inner ear cell types, we can investigate the role of different mitochondrial configurations in cell fate decisions, functional refinement and during cell death in the epithelia of inner ear sense organs. With a better understanding of how mitochondrial activity regulates HC death, we also can work towards developing therapeutic tools to preserve these cells during various treatment regimes.

## CRediT authorship contribution statement

**James D.B. O'Sullivan:** Writing – original draft, Writing – review & editing. **Anwen Bullen:** Writing – original draft, Writing – review & editing, Visualization. **Zoë F. Mann:** Writing – original draft, Writing – review & editing.

## Declarations of Competing Interest

None.

## Data Availability

The authors are unable or have chosen not to specify which data has been used.
